# Role of the Contralesional vs. Ipsilesional Hemisphere in Stroke Recovery

**DOI:** 10.3389/fnhum.2017.00469

**Published:** 2017-09-21

**Authors:** Keith C. Dodd, Veena A. Nair, Vivek Prabhakaran

**Affiliations:** ^1^Department of Biomedical Engineering, University of Wisconsin-Madison Madison, WI, United States; ^2^Department of Radiology, School of Medicine and Public Health, University of Wisconsin-Madison Madison, WI, United States; ^3^Medical Scientist Training Program, School of Medicine and Public Health, University of Wisconsin—Madison Madison, WI, United States; ^4^Neuroscience Training Program, University of Wisconsin—Madison Madison, WI, United States; ^5^Department of Neurology, University of Wisconsin—Madison Madison, WI, United States; ^6^Department of Psychology and Department of Psychiatry, University of Wisconsin—Madison Madison, WI, United States

**Keywords:** ipsilesional, contralesional, stroke, motor recovery, brain-computer interface, transcranial magnetic stimulation

## Abstract

Following a stroke, the resulting lesion creates contralateral motor impairment and an interhemispheric imbalance involving hyperexcitability of the contralesional hemisphere. Neuronal reorganization may occur on both the ipsilesional and contralesional hemispheres during recovery to regain motor functionality and therefore bilateral activation for the hemiparetic side is often observed. Although ipsilesional hemispheric reorganization is traditionally thought to be most important for successful recovery, definitive conclusions into the role and importance of the contralesional motor cortex remain under debate. Through examining recent research in functional neuroimaging investigating motor cortex changes post-stroke, as well as brain-computer interface (BCI) and transcranial magnetic stimulation (TMS) therapies, this review attempts to clarify the contributions of each hemisphere toward recovery. Several functional magnetic resonance imaging studies suggest that continuation of contralesional hemisphere hyperexcitability correlates with lesser recovery, however a subset of well-recovered patients demonstrate contralesional motor activity and show decreased functional capability when the contralesional hemisphere is inhibited. BCI therapy may beneficially activate either the contralesional or ipsilesional hemisphere, depending on the study design, for chronic stroke patients who are otherwise at a functional plateau. Repetitive TMS used to excite the ipsilesional motor cortex or inhibit the contralesional hemisphere has shown promise in enhancing stroke patients' recovery.

## Introduction

Stroke remains a leading cause of long-term disability (Hoyer and Celnik, 2011), affecting nearly 800,000 people annually within the United States (Mozaffarian et al., 2016), and causing almost 80% of patients to have persistent motor impairment following traditional therapy (Mayo et al., 1999). Imaging assessment of neural injury, function, and stroke subtype appear to have significant effects on stroke recovery (Burke Quinlan et al., 2015). Specifically, the initial degree of motor impairment (Byblow et al., 2015) and degree of injury to the corticospinal tract (Burke Quinlan et al., 2015; Feng et al., 2015; Doughty et al., 2016) have shown to be strong predictors of recovery, and several chronic stroke patients demonstrate functional recovery plateaus following standard rehabilitation (Young et al., 2014a).

Initial stroke recovery correlates to resolution of necrotic tissue, edema, and inflammation (Furlan et al., 1996; Stinear and Byblow, 2014), while later recovery relates mainly to disinhibition of redundant neural circuits, recruitment of functionally homologous pathways, and the creation of neural connections to overtake the previous functions of the damaged neurons (Rossini et al., 2007; Murphy and Corbett, 2009; Ackerley et al., 2011). Such neuronal processes may occur on the ipsilesional and contralesional hemispheres and are not completely understood (Hoyer and Celnik, 2011; Buetefisch, 2015); therefore, the relationship between the two motor cortex (M1) hemispheres post-stroke remains a topic of great interest (Baron et al., 2004; Hummel et al., 2008).

**Figure 1:**
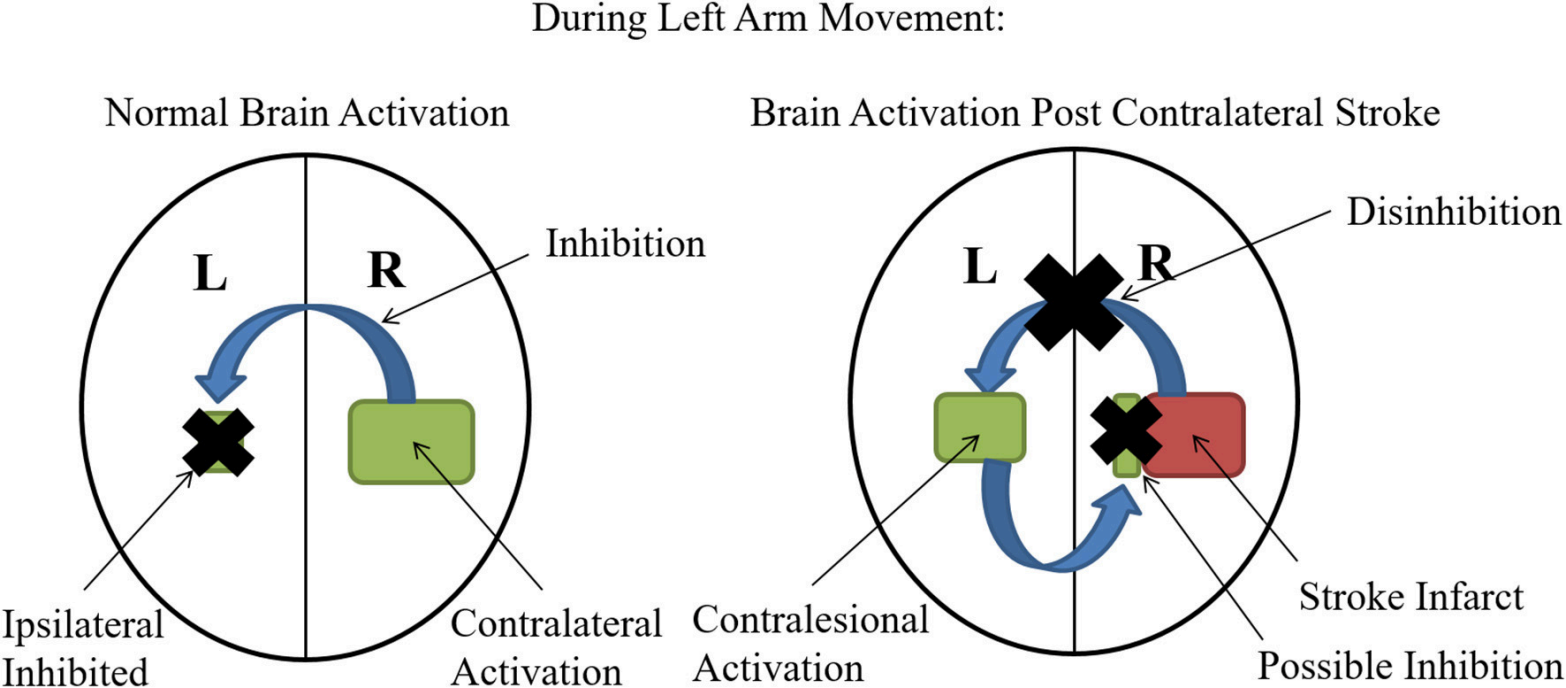
Interhemispheric Imbalance Post Stroke: Normally during unilateral motor performance tasks, activation of the contralateral hemisphere inhibits activation of the ipsilateral hemisphere (black X is showing inhibition of the ipsilateral side). However, when the contralateral M1 is impacted by stroke, this inhibition is lost (black X on the blue inhibitory arrow) and therefore the contralesional (analogous to ipsilateral for healthy controls) becomes more activated. Some studies suggest that this increased contralesional activation may also contribute to the interhemispheric imbalance by imposing increased inhibition on the ipsilesional (contralateral for healthy patients) hemisphere designated by the blue arrow and black X on the ipsilesional hemisphere (Murase et al., 2004).

The contralateral M1 normally inhibits the ipsilateral hemisphere during motor performance tasks (Ocklenburg et al., 2015). However, patients with recent stroke commonly demonstrate increased M1 excitability on the contralesional hemisphere (equivalent to the ipsilateral hemisphere for healthy patients) for movements with the affected side (Chollet et al., 1991; Weiller et al., 1992; Shimizu et al., 2002; Butefisch et al., 2003, 2008; Murase et al., 2004; Tang et al., 2015). Therefore, stroke patients display an interhemispheric imbalance where the ipsilesional M1 no longer inhibits the contralesional hemisphere and the contralesional side appears to inhibit the ipsilesional (Baron et al., 2004), possibly through the transcallosal fibers (Murase et al., 2004) (Figure 1). The magnitude of such an imbalance appears to positively correlate with the degree of motor impairment (Murase et al., 2004), and the interhemispheric imbalance in other functional networks may also contribute toward other cortical functional disruptions including neglect (Tomaiuolo et al., 2010) and aphasia (Griffis et al., 2016).

Therapies that offer repeated action of the impaired arm, including mirror therapy (MT) (Rossiter et al., 2015), virtual-reality therapy (VRT) (Laver et al., 2015), constraint-induced movement therapy (CIMT) (Kwakkel et al., 2015), and brain-computer interface (BCI) therapy may reverse progressive down-regulation of sensorimotor activity (Lindberg et al., 2007) and induce neural plasticity (Johnson et al., 2004). However, most current rehabilitation methods that can target a specific M1 hemisphere, such as, transcranial magnetic stimulation (TMS), focus on ipsilesional hemispheric activation to facilitate motor recovery (Sanes et al., 1990; Hoyer and Celnik, 2011; Buetefisch, 2015). Although several studies demonstrate poorer motor recovery with increased contralesional hemispheric recruitment (Loubinoux et al., 2003; Fridman et al., 2004; Ward and Cohen, 2004; Calautti et al., 2007), some evidence suggests that contralesional recruitment may play an integral role in post-stroke motor recovery. According to Hoyer et al., the importance and role of contralesional M1 neural activity is probably determined by variables including time since stroke, degree of damage to the motor system, and complexity of the motor task (Hoyer and Celnik, 2011).

Brain-computer interfaces are a newer modality that use reward-based neuromodulatory training to induce use-dependent plasticity and facilitate functional recovery for stroke rehabilitation (Li et al., 2014; Young et al., 2014b; Soekadar et al., 2015; Mrachacz-Kersting et al., 2016). Unlike MT, VRT, CIMT, and TMS, BCI therapy uses signal detection of neural activity through an electroencephalogram (EEG) to provide specific and immediate visual feedback to the patient depending on their performance (Buch et al., 2008; Young et al., 2014a). This mini review attempts to broaden the scientific community's understanding of the roles of the M1 hemispheres in stroke rehabilitation by: (1) Analyzing current research on the role of the M1 hemispheres for spontaneous stroke recovery, and (2) Examining results from a therapy that relies upon specific M1 neuronal signals for feedback (BCI) as well as therapy that targets specific M1 hemispheres (TMS).

## The role of the contralesional and ipsilesional hemispheres for spontaneous stroke recovery

Most stroke patient brain activation studies find increased bilateral activation, related to elevated contralesional M1 activation, correlates to poorer motor recovery (Loubinoux et al., 2003; Baron et al., 2004). For example, increases in ipsilesional M1 activation, indicative of lesser bilateral activation, have significantly predicted larger treatment-induced behavioral gains for patients with lacunar infarcts (Burke Quinlan et al., 2015). Meanwhile, Ward et al.'s (2003a) cross-sectional study demonstrates a shift from bilateral activation to unilateral ipsilesional M1 activation in the more well-recovered patients during movement of the affected hand (Ward et al., 2003a) with other cross-sectional studies showing similar results (Chollet et al., 1991; Ward et al., 2003b). Longitudinal studies have also shown this trend with increased recovery correlated to increased unilateral development (Ward et al., 2003b). A limitation of many of these studies was significant mirror movements of the unaffected hand (Nelles et al., 1999, 2001; Carey et al., 2002; Feydy et al., 2002; Small et al., 2002). Such movements, indicative of poor motor outcome (Nelles et al., 1998; Kim et al., 2003), could have contributed to greater contralesional M1 activity (Buetefisch, 2015). Rodent stroke models that display decreased motor recovery when the non-affected limb is trained further suggests that increased contralesional M1 activation hinders motor recovery (Allred et al., 2005, 2010; Allred and Jones, 2008). In a recent systematic meta-analysis of studies in human stroke patients, Tang et al. demonstrated increased interhemispheric balance activation in patients within the sensorimotor and premotor cortices in well-recovered stroke patients (Tang et al., 2015). To facilitate optimal motor recovery, one might attempt to re-establish a normalized hemispheric balance between the two sides of the motor cortex given the evidence that activation for most well-recovered stroke patients shifts from a bilateral to an unilateral pattern (Hoyer and Celnik, 2011).

Increased ipsilateral M1 activation has been shown for tasks with higher accuracy or complexity demands in healthy subjects (Winstein et al., 1997; Hummel et al., 2003; Seidler et al., 2004; Verstynen et al., 2005; Buetefisch et al., 2014). Additionally, well-recovered stroke patients with chronic striatocapsular motor strokes (≥2 months post-stroke) show notable contralesional motor activity (Weiller et al., 1992), and Schaechter and Perdue demonstrated that increased activation of the contralesional cortical network in chronic stroke (≥6 months post-stroke) patients is associated with good motor recovery (Schaechter and Perdue, 2008). These findings suggest that uncrossed corticospinal tracts, which include roughly 10% of the corticospinal neuronal fibers, play a role in motor movement and possibly in recovery (Brus-Ramer et al., 2009). Touvykine et al. suggests that the contralesional premotor cortex may play a greater role in stroke recovery with larger lesions where damage on the ipsilesional side is more severe and where less ipsilesional neuronal recovery can develop (Touvykine et al., 2016). This indicates that activation of the contralesional hemisphere provides recruitment of additional neural resources due to the increased demands of the damaged ipsilesional motor system (Riecker et al., 2010). While some research suggests that increased activation of the contralesional M1 may detract from motor recovery (Calautti and Baron, 2003; Ward et al., 2003b), recent evidence suggests continued supportive functions of the contralesional hemisphere in a subset of chronic stroke patients (Butefisch et al., 2005; Lotze et al., 2006; Riecker et al., 2010). As most motor-impaired stroke patients initially demonstrate increased contralesional M1 activation (Murase et al., 2004), it is possible this activation offers increased motor function benefits (Lotze et al., 2006) within the subacute stage, but may interfere with continued recovery for some patients (Mansur et al., 2005; Fregni et al., 2006). Regardless, the contralesional M1 appears to support the function of the impaired limb in a subset of chronic stroke patients (Lotze et al., 2006), depending on factors including lesion location and size, but it may interfere in complete recovery of the limb in other subsets (Mansur et al., 2005; Fregni et al., 2006).

TMS can index interhemispheric interactions in patients >14 months post stroke (Borich et al., 2016) and has been used to help characterize the roles of the contralesional and ipsilesional hemispheres in spontaneous stroke recovery. Multiple studies utilizing TMS suggest that continuation of the increased activation of contralesional M1 associates with lesser clinical outcome (Turton et al., 1996; Netz et al., 1997; Feydy et al., 2002; Johansen-Berg et al., 2002). For instance, for 12 individuals with major arm paresis post-stroke, inhibitory TMS of the contralesional M1 significantly increased movement time in a paretic arm reaching task suggesting that patients with severe arm impairment rely heavily on the contralesional hemisphere (Mohapatra et al., 2016). Stinear et al. demonstrates that ipsilesional corticomotor excitability increased with time during recovery of 46 patients (44 with subcortical damage, and only two with motor cortex damage) during the first 6-months post-stroke (Stinear et al., 2015). However, interhemispheric inhibition of these patients was shown to be stable and symmetrical over time, with no decrease in contralesional corticomotor excitability (Stinear et al., 2015).

Other TMS studies suggest that contralesional hemispheric recruitment is a factor of well-recovered stroke patients. For instance, Lotze et al. demonstrated that rTMS applied to the contralesional hemisphere in well-recovered patients induced timing and accuracy deficits during production of a complex sequential motor task (Lotze et al., 2006). Furthermore, although TMS of only the ipsilesional hemisphere of well-recovered stroke patients, and not the contralesional, induced motor-evoked potentials in Nair et al., TMS did reveal greater transcallosal inhibition from the contralesional to the ipsilesional hemisphere than in the reverse direction (Nair et al., 2007). In Julkunen et al., TMS in conjunction with diffusion weighted imaging revealed that inter-hemispheric asymmetry in the M1 persists even years after stroke for well-recovered patients where clinical symptoms have normalized (Julkunen et al., 2016). These findings, along with a number of noninvasive brain stimulation studies that report null effects, suggest that the aim to strictly normalize the interhemispheric balance may be over-simplistic (Di Pino et al., 2014). Instead, other more complex models, such as, a bimodal balance-recovery model that links interhemispheric balancing and functional recovery to the amount of structural reserve spared by the lesion, may prove more accurate at determining optimal therapy for stroke patients (Di Pino et al., 2014).

## The role of the contralesional and ipsilesional hemispheres with newer modality therapy (BCI & TMS)

Some BCI paradigms have demonstrated motor improvements associated with recovery of the contralesional hemisphere. The BCI design in Song et al. and Young et al. demonstrated that increased contralesional hemispheric activation correlated to better performance on the Nine-Hole Peg Test (9-HPT), Action Research Arm Test (ARAT), and Stroke Impact Scale (SIS) (Song et al., 2014; Young et al., 2014a). Additionally, contralesional corticospinal tract (CST) fractional anisotropy (FA) correlated positively with improvements in the 9-HPT and increases in asymmetric FA correlated with the ARAT (Young et al., 2016). Similarly, Bundy et al.'s BCI, which focused on contralesional motor activation, found significant increases in the ARAT, grasp strength, Motricity Index, and the Canadian Occupational Performance Measure (Bundy et al., 2017). Young's, Song's, and Bundy's BCI paradigms suggested that axonal remodeling within, and increased motor fiber integrity of, the contralesional hemisphere correlates with individual motor gains for patients with upper-limb impairment (Song et al., 2014; Young et al., 2014a; Bundy et al., 2017).

In contrast, other BCI paradigms display motor improvements correlated to recovery of the ipsilesional hemisphere. Ramos-Murguialday et al.'s BCI [denoted as a brain-machine-interface (BMI)], saw significant motor gains associated with increased ipsilesional M1 recruitment with their experimental group as compared to control (Ramos-Murguialday et al., 2013). Additionally, only the experimental group demonstrated significantly improved modified upper limb Fugel-Meyer Motor Assessment (FMA) scores. Similarly, in Pichiorri et al., BCI training was associated both with increased ipsilesional hemisphere activation in response to motor imagery of the paralyzed limb and increased functional outcome in the FMA (Pichiorri et al., 2015).

Reasons for the differences found in the BCI studies probably relate to the BCI/BMI designs and the patient exclusion criteria. For instance, Young and Song recruited patients with a greater range of severity of motor impairments than Ramos (Ramos-Murguialday et al., 2013; Young et al., 2016). Additionally, Ramos' BMI paired orthoses movements with desynchronization of ipsilesional hemisphere brain oscillations (Ramos-Murguialday et al., 2013), Young and Song rewarded μ (8–12 Hz) and β (18–26 Hz) rhythms with functional electrical stimulation (FES), tongue display unit stimulation, and visual ball movement (Song et al., 2014; Young et al., 2014a), and Bundy used a moving hand exoskeleton that responded to contralesional motor signals (Bundy et al., 2017).

Overall, multiple BCI studies have found improved motor function for the severely impaired stroke patient correlated with contralesional M1 recruitment (Song et al., 2014; Young et al., 2014a; Bundy et al., 2017) as well as ipsilesional M1 recruitment (Ramos-Murguialday et al., 2013; Pichiorri et al., 2015). Specific BCI designs, as well as patient recruitment qualifications probably determine which hemisphere will activate more to begin motor function compensation development. Wide variation in the behavioral assessments used for outcome evaluation of motor function after stroke may also contribute to the heterogeneity in results making direct comparisons between study results challenging (Broetz et al., 2014). However, with the meta-analysis by Lohse et al. demonstrating that the trend of increased therapy correlating with increased improvement is not altered by stroke chronicity (Lohse et al., 2014), BCI appears to be a promising therapy for stroke patients who have reached a functional plateau with traditional therapy (Young et al., 2015).

TMS therapy can inhibit or excite targeted brain regions, and applications of this technique may encourage stroke rehabilitation (Hallett, 2000; Fregni et al., 2006) without deteriorating motor performance in the non-affected hand (Fregni et al., 2006; Vines et al., 2008; Hoyer and Celnik, 2011). One therapeutic utilization of TMS includes inhibitory, low-frequency, rTMS trains onto the contralesional motor region (Kobayashi et al., 2004). Numerous studies have already found improved task performance on the affected side following contralesional M1 inhibition via rTMS applied at a low-frequency (Demirtas-Tatlidede et al., 2015; Matsuura et al., 2015; Ludemann-Podubecka et al., 2016). Kobayashi et al. demonstrated that for healthy subjects, inhibitory 1-Hz rTMS over the non-performing M1 shows improvement of motor task performance of the ipsilateral hand, possibly by enhancing cortical excitability of the ipsilesional M1 through decreased interhemispheric inhibition from the stimulated (Kobayashi et al., 2004; Grefkes et al., 2010). Additionally, regional cerebral blood flow in the left M1 during right hand movement has been seen to increase during 1-Hz inhibitory rTMS of the right M1 (Conchou et al., 2009). For a study with eight mild-to-moderate stroke patients (<1 year post stroke), there was modest improvement in their motor function following an inhibitory rTMS applied to the contralesional hemisphere (Mansur et al., 2005). In a 2015 study, stroke patients with mild to moderate upper limb motor impairment were either subjected to 1-Hz contralesional M1 inhibitory rTMS or sham rTMS. Results demonstrated that a 1-Hz rTMS 15-day treatment plan showed significant motor dexterity improvement in dominant hemisphere strokes, but not those with strokes in the non-dominant hemispheres (Ludemann-Podubecka et al., 2015). In contrast Rose et al. found no significant improvement in restoration of upper extremity functional use for post 6-month stroke patients with a four-times-a-week program of inhibitory rTMS for 4 weeks (Rose et al., 2014). This study suggests that increased chronicity of stroke causes impairment of beneficial effects of rTMS in motor recovery (Rose et al., 2014).

Another method to regain interhemispheric balance and improve recovery is to excite the ipsilesional side with TMS (Talelli et al., 2007; Ameli et al., 2009). Kim et al. found that high-frequency rTMS to the motor cortex ipsilesionally gave improved finger sequence performance in the contralateral hand (Kim et al., 2004). Additionally, Khedr found that acute stroke patients with excitatory 3-Hz rTMS over the ipsilesional M1 for 10 days showed improved motor function (Khedr et al., 2005). Du et al. demonstrated that 3-Hz ipsilesional M1 rTMS showed greater motor improvements sustained for at least 3 months post treatment as compared to a control group with sham rTMS (Du et al., 2016). Brodie et al. confirmed that 5-Hz rTMS ipsilesionally enhances motor learning for chronic stroke (>6 months post) patients as compared to a sham control group over a serial tracking task (Brodie et al., 2014). Specifically the experimental group demonstrated significantly greater improvements in peak velocity, cumulative distance, and cutaneous somatosensation. The study concludes that when paired with motor practice, as both groups also went under tracking task training, 5-Hz excitatory rTMS over the ipsilateral M1 enhances motor learning in chronic stroke patients although no improvement in general upper extremity function was observed (Brodie et al., 2014).

A few studies have compared inhibiting the contralesional hemisphere to exciting the ipsilesional side through rTMS although the results remain inconclusive. Khedr et al. (2009) compared 1-Hz rTMS inhibition to 3-Hz rTMS ipsilesional M1 stimulation on simple motor tasks including keyboard tapping and a nine-hole pegboard task. This study found that the contralesional hemisphere 1-Hz inhibitory pulse sequence showed more motor improvement (Khedr et al., 2009). Similarly, another study compared 1 Hz inhibition contralesionally to 10-Hz excitatory ipsilesionally and a bilateral combination of both with the conclusion that only contralesional rTMS and bilateral showed significant improvement in motor training lasting 1 week (Takeuchi et al., 2009). Despite these findings, Emara et al. compared 1-Hz contralesional stimulation to 5-Hz ipsilesional hemisphere stimulation over 10 days and found that both group had significant motor function and disability scale improvement which lasted up to 12 weeks (Emara et al., 2010). Kim et al. also did not measure significant differences in motor function of the upper extremity between 1-Hz inhibitory and 20-Hz excitatory treatment for patients who suffered from their first subacute stroke within 4 weeks of the study (Kim et al., 2014). Finally, Mcdonnell and Stinear's meta-analysis found no clear evidence of interhemispheric inhibition or hyper-excitability of the unaffected hemisphere suggesting that exciting the ipsilesional M1 with TMS may be more beneficial than inhibiting the contralesional M1 (McDonnell and Stinear, 2017).

## Conclusion

Major factors influencing unilateral recovery post-stroke appear to include chronicity, as well as lesion size and location. Increases in unilateral ipsilesional M1 activation for movement on the affected side appears to positively correlate with chronicity, especially for well-recovered patients (Ward et al., 2003b). However, increased contralesional M1 recruitment has been demonstrated for well-recovered patients with larger lesions (Touvykine et al., 2016). Overall, well-recovered patients display increased unilateral activation of the ipsilesional M1 with motor tasks as compared to ill-recovered patients, and animal models have suggested that motor learning of the non-affected side during recovery could limit rehabilitation of the ipsilesional hemisphere (Allred et al., 2005, 2010; Allred and Jones, 2008; Jones et al., 2013). However, some well-recovered patients display notable contralesional motor activity and the contralesional hemisphere appears to play a key role for at least a subset of stroke patients.

BCI training demonstrates potential in assisting with motor recovery for stroke sufferers who have reached a functional plateau following traditional rehabilitation. Evidence suggests that this therapy, even for chronic stroke sufferers, may induce neuromodulatory changes that enhance motor function. Both BCI designs that demonstrate development of the contralesional M1, as well as designs that increase ipsilesional M1 activity, appear to enhance motor recovery. It is currently unclear if either type of BCI design is superior to the other for all, or certain subsets of, patients.

Meanwhile, TMS is an effective and safe tool for research into this field and offers promising potential to be utilized for rehabilitation. TMS studies confirm that increased contralesional M1 activity as compared to the ipsilesional M1 is associated with lesser clinical outcome. Some TMS research suggests that for some well-recovered stroke patients contralesional corticomotor excitability does not decrease, but rather only the ipsilesional corticomotor excitability increases with recovery (Stinear et al., 2015) and often the interhemispheric asymmetry within M1 persists even years after stroke (Julkunen et al., 2016).

Both inhibition of the contralesional side and excitation of the ipsilesional side appear effective for motor recovery post-stroke. A few studies suggest that contralesional inhibition may be slightly more effective than ipsilesional M1 excitation although this is not seen throughout the literature. Initial degree of motor impairment and chronicity (Rose et al., 2014), as well as age (Kim et al., 2016), have been suggested to negatively correlate with the ability for rTMS to enhance the recovery process.

Current evidence favors ipsilesional M1 excitation and development to be the most important for stroke recovery over contralesional hemispheric recruitment. However, the contralesional side does appear to play a significant role for at least a subset of stroke patients and some research suggests that certain BCI paradigms targeting the contralesional hemisphere may be beneficial for motor recovery. It is important to note the numerous confounding variables that require more research to completely understand their effects including lesion location and size, and chronicity.

## Author contributions

KD wrote the manuscript, VN and VP reviewed and edited it, and provided key guidance.

### Conflict of interest statement

VP has a pending US patent on the closed-loop neurofeedback used for BCI-facilitated intervention, application number 12/715090. The patent was filed jointly by VP and J. Williams. The other authors declare that the research was conducted in the absence of any commercial or financial relationships that could be construed as a potential conflict of interest.
